# Epibulbar complex choristoma with simultaneous involvement of eyelid: a case report

**DOI:** 10.1186/s12886-019-1234-7

**Published:** 2019-11-12

**Authors:** Yun Hsia, Huang-Chun Lien, I-Jong Wang, Shu-Lang Liao, Yi-Hsuan Wei

**Affiliations:** 1 Department of Ophthalmology, National Taiwan University Hospital, No 7, Chung-Shan S. Rd, Taipei 100, Taiwan; 2 Department of Pathology, National Taiwan University Hospital, Taipei, Taiwan; 3 College of Medicine, National Taiwan University, Taipei, Taiwan

**Keywords:** Epibulbar complex choristoma, Eyelid tumor, Congenital tumor, Case report

## Abstract

**Background:**

Epibulbar complex choristoma, a rare congenital epibulbar tumor, has many diverse forms. Reviewing the literature, it can present clinically as either a circumferential or isolated epibulbar mass, limbal tumor, lateral canthal mass, aggregate of ectopic cilia in the upper eyelid, eyelid mass mimicking chalazion, or lacrimal caruncle mass. The management depends on the extent of involvement, the risk of amblyopia, and cosmetic concerns. Here, we report an atypical presentation of epibulbar complex choristoma with simultaneous eyelid involvement.

**Case presentation:**

A 1-month-old full-term boy was brought to our clinic with congenital epibulbar mass of the right eye with simultaneous eyelid involvement. Dilated fundus examination was unremarkable. Survey for linear nevus sebaceous Jadassohn was negative. Due to concerns of possible amblyopia and cosmetics, lamellar keratectomy, sclerotomy, and conjunctivoplasty were performed to remove the epibulbar lesion. The eyelid defect was reconstructed with 6–0 Vicryl sutures. Histopathological examination reported complex choristoma. Upon three-year follow-up, low astigmatism and favorable cosmetics results were achieved.

**Conclusions:**

Congenital complex choristoma can present clinically as an epibulbar mass with eyelid involvement. The management depends on the extent of involvement, the risk of amblyopia, and cosmetic concerns. The method of eyelid reconstruction should be tailored according to the residual eyelid defect.

## Background

Epibulbar choristoma represents a histologically normal tissue in an abnormal location, with reported incidence between 1/10000 and 1/30000 [1, 2]. Limbal dermoid, the most common type, is characterized by well-circumscribed, keratinized, dome-shaped, inferotemporally located limbal mass [2]. Complex choristoma, another subgroup of choristoma, consists of tissue from two or more origins [1]. In comparison to limbal dermoid, complex choristoma is rarely reported in the literature and has diverse clinical presentations [1–7], including circumferential or isolated epibulbar mass [1, 3], limbal tumor with corneal involvement [4], lateral canthal mass [3], aggregate of ectopic cilia in the upper eyelid [5], eyelid mass mimicking chalazion [6], or lacrimal caruncle mass [7]. Reporting the previously unseen presentation can help clinicians to gain greater understanding of the pathology and characteristics of this rare disease. Here, we present a unique case of complex choristoma with simultaneous epibulbar and eyelid involvement, which has not been previously reported.

## Case presentation

**Fig. 1:**
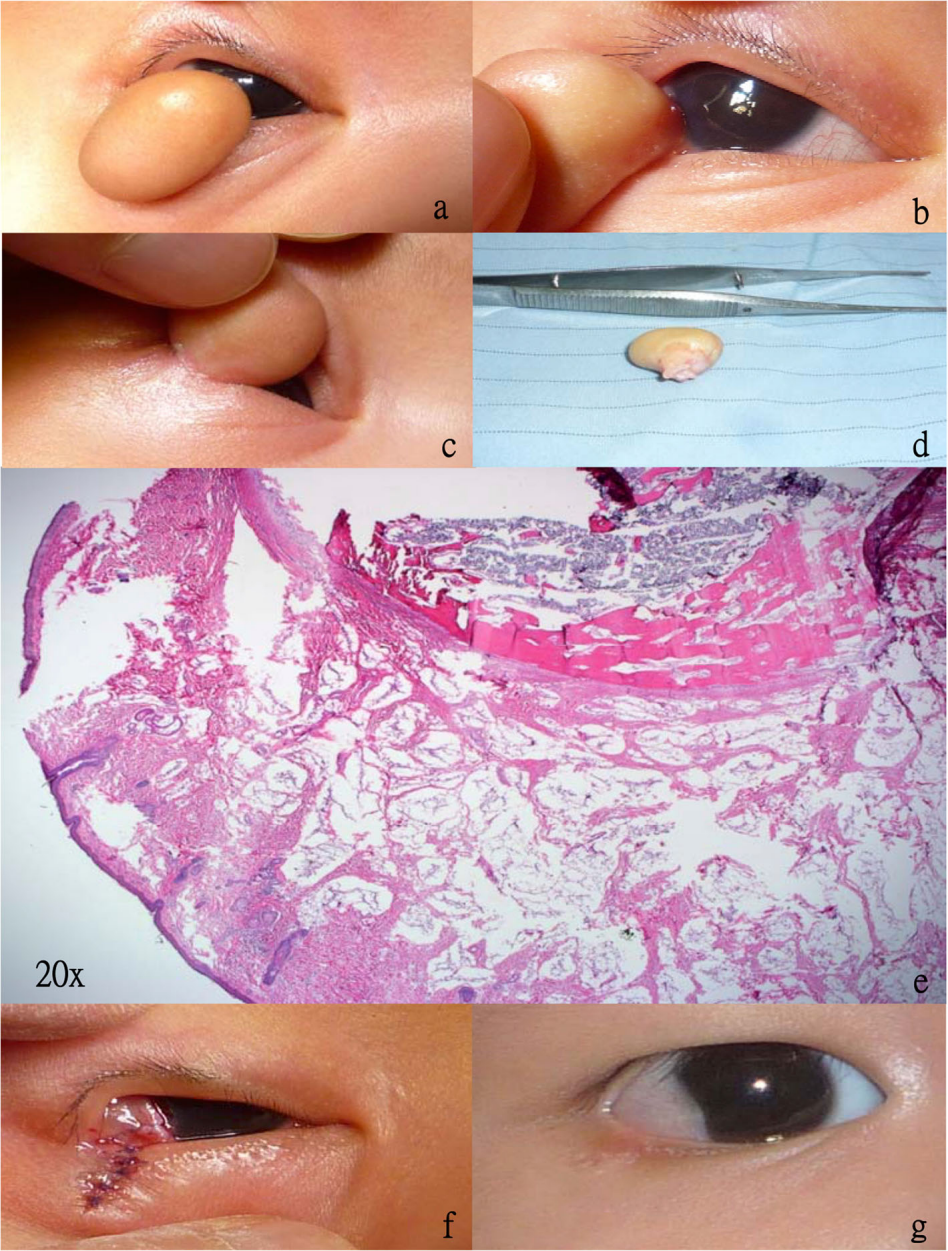
Clinical and histopathological manifestation of complex choristoma. **a** The bulky epibulbar lesion blocked the visual axis. **b** Adherence of the epibulbar lesion to the limbal area with minimal corneal involvement. **c** Simultaneous involvement of the lower lid margin by the mass lesion. **d** Removal of the mass lesion, with the conjunctival excision surface revealed in this photo. **e** Microscopically, the mass lesion consisted of mature adipose tissue and bony trabeculae with marrow components. **f** Closure of the eyelid defect by sutures, and covering of the bare sclera with conjunctiva after removal of the mass lesion. **g** Smooth lower lid margin 3 months after the operation, with a pseudopterygium noted at the temporal limbal area

A 1-month-old boy was presented to our clinic with a bulky bulging mass in the right eye since birth. He was delivered following an uneventful full-term pregnancy with no particular perinatal insults. Although formal visual acuity measurements could not be obtained, the patient’s gaze could fix and follow and was able to achieve central, steady, and maintained fixation. Physical examination noted the bulging epibulbar mass extending out from his right eye to his eyelid (Fig. 1 a-c). No further ocular nor systemic abnormalities were identified, and no significant ocular motility limitation was detected. Dilated fundus examination was unremarkable. Under the clinical impression of choristoma, lamellar keratectomy and sclerotomy were performed to remove the epibulbar lesion, for cosmetics purposes and to alleviate the risk of amblyopia. The eyelid lesion was removed by excision and the defect was reconstructed with 6–0 Vicryl sutures. The defect of ocular surface could be fully reconstructed by conjunctivoplasty; therefore, amniotic membrane transplantation was not performed. The pathological report measured a 1.7*1.4*1.2 cm elastic mass under gross inspection (Fig. 1d). Microscopically, the mass lesion consisted of mature adipose tissue and bony trabeculae with marrow components, compatible with complex choristoma (Fig. 1e). Topical gentamycin, fluorometholon eye drops four times per day and Maxitrol ointment once at bedtime were given post-operatively for 2 weeks. Three months after the surgery, the eyelid margin was smooth and the ocular surface was silent apart from a pseudopterygium on the temporal side (Fig. 1 f, g). Minimal astigmatism of 0.25D, suggests lowered risk for future amblyopia development. Three years after the surgery, the pseudopterygium persisted and the astigmatism was stable.

## Discussion and conclusions

Choristoma, histologically normal tissue in an abnormal location, represents 10–33% of conjunctival lesions in children. Limbal dermoid and dermolipoma are the most common type [8]. Complex choristomas are characterized by containing two or more ectopic tissues, which may include bone, cartilage, lacrimal gland tissue, muscle, nerve and adipose tissues [1, 4, 9]. In clinical practice, epibulbar complex choristomas are rarely seen, with diverse reported presentations, including circumferential or isolated epibulbar mass [1, 3], limbal tumor with corneal involvement [4], lateral canthal mass [3], aggregate of ectopic cilia in the upper eyelid [5], eyelid mass mimicking chalazion [6], or lacrimal caruncle mass [7].

Most of the complex choristoma reported in literature were covered by mucous membrane [1, 6, 10]. A recent study identified complex cartilaginous choristoma covered by pterygium [9]. Even though the skin-covered surface and inferotemporal location in our case are reminiscent of limbal dermoid, the pathology report revealed complex choristoma. Due to the variable presentations of complex choristomas, diagnosis can only be confirmed with histopathological examination.

The association between epibulbar complex choristoma and linear nevus sebaceous of Jadassohn has been well-established. The latter is characterized by seizures, mental retardation, and cutaneous sebaceous nevus of the facial and scalp area [1], as well as possible ocular manifestations of coloboma of the eyelid, iris, choroid, and optic disc, other optic disc abnormalities, choroidal osteoma, light-colored fundus lesion, and oculomotor nerve palsy [4]. Histopathologically confirmed complex choristoma should prompt the survey for co-existence of linear nevus sebaceous of Jadassohn, which should negative results in our patient.

Due to complex choristomas generally regarded as benign and non-progressive lesions, the indications of treatment are mainly for cosmetic purpose or for amblyopia prevention [10]. However, slow growth or even rapid progression has been reported, often associated with linear nevus sebaceous of Jadassohn [11, 12]. Early surgical intervention should be considered in these cases. Treatment should be planned according to the depth, site and size of the mass lesion, and the extent of involvement, ranging from simple excision, amniotic membrane transplantation, lamellar keratoplasty, penetrating keratoplasty to autologous limbal stem cell allograft [10]. Full-thickness central corneal grafts can be utilized to achieve good cosmetic results in lamellar keratoplasty [13]. In cases with large and deep defect after excision of the mass lesion, scleral graft or corneal graft can be used to reconstruct the ocular surface [14]. In cases with simultaneous eyelid involvement, eyelid reconstruction should be tailored according to the residual eyelid defect. In our case, the eyelid defect was repaired with primary closure, while conjunctivoplasty was performed to cover the scleral and corneal defect. Favorable cosmetic outcome was achieved after the surgery.

The risk of amblyopia depends on the extent of corneal and visual axis involvement. Close postoperative follow-up is necessary to identify and prevent possible deprivation or refractive amblyopia [1]. Given that our patient suffered minimal corneal involvement and received early surgical intervention, the risk for future amblyopia development is considered low. In conclusion, epibulbar complex choristoma can have simultaneous eyelid involvement. Timely and tailored surgical intervention can achieve satisfying cosmetic and visual outcome.

## Data Availability

Data sharing is not applicable to this article as no datasets were generated or analyzed during the current study.
